# Initial Gamma Knife Radiosurgery for Large or Documented Growth Asymptomatic Meningiomas: Long-Term Results From a 27-Year Experience

**DOI:** 10.3389/fonc.2020.598582

**Published:** 2020-11-24

**Authors:** Junyi Fu, Lisha Wu, Chao Peng, Xin Yang, Hongji You, Linhui Cao, Yinhui Deng, Jinxiu Yu

**Affiliations:** ^1^ Department of Neurology, The Second Affiliated Hospital of Guangzhou Medical University, Guangzhou, China; ^2^ Department of Medical Oncology, Sun Yat-sen Memorial Hospital, Sun Yat-sen University, Guangzhou, China; ^3^ Department of Neurosurgery, Guangdong Provincial People’s Hospital, Guangdong Academy of Medical Sciences, Guangzhou, China; ^4^ Department of Thoracic Surgery, The Second Affiliated Hospital of Guangzhou Medical University, Guangzhou, China; ^5^ Department of Nuclear Medicine, The Second Affiliated Hospital of Guangzhou Medical University, Guangzhou, China; ^6^ Department of Traditional Chinese Medicine, Sun Yat-sen Memorial Hospital, Sun Yat-sen University, Guangzhou, China; ^7^ Department of Radiotherapy, The Second Affiliated Hospital of Guangzhou Medical University, Guangzhou, China

**Keywords:** peritumoral edema, gamma knife, stereotactic radiosurgery, asymptomatic meningioma, progression-free survival

## Abstract

**Objective:**

The aims of this study were to investigate the long-term outcomes of initial Gamma Knife radiosurgery (GKRS) for large (≥20 mm) or documented growth asymptomatic meningiomas.

**Design and Methods:**

This was a single-center retrospective study. Fifty-nine patients with large (≥20 mm) or documented growth asymptomatic meningiomas undergoing initial GKRS were enrolled. The median age was 56 (range, 27–83) years. The median time of follow-up was 66.8 (range, 24.6–245.6) months, and the median tumor margin dose was 13.0 Gy (range, 11.6–22.0 Gy).

**Results:**

Tumors shrunk in 35 patients (59.3%) and remained stable in 23 (39.0%). One patient (1.7%) experienced radiological progression at 54 months after GKRS. The PFS was 100%, 97%, and 97% at 3, 5, and 10 years, respectively. Nine patients (15.3%) occurred new neurological symptoms or signs at a median time of 8.1 (range, 3.0–81.6) months. The symptom PFS was 90% and 78% at 5 and 10 years, respectively. Fifteen patients (25.4%) occurred peritumoral edema (PTE) at a median time of 7.2 (range, 2.0–81.6) months. One patient underwent surgical resection for severe PTE. In univariate and multivariate analysis, Only tumor size (≥25 mm) and maximum dose (≥34 Gy) were significantly associated with PTE [hazard ratio (HR)= 3.461, 95% confidence interval (CI)=1.157-10.356, p=0.026 and HR=3.067, 95% CI=1.068-8.809, P=0.037, respectively].

**Conclusions:**

In this study, initial GKRS can provide a high tumor control rate as well as an acceptable rate of complications in large or documented growth asymptomatic meningiomas. GKRS may be an alternative initial treatment for asymptomatic meningiomas.

## Introduction

Meningiomas are the most common intracranial benign tumors, which represent almost 13%–37.6% of all intracranial tumors (1–4). Of those meningiomas with documented WHO grade, 80.5% are grade I, 17.7% are grade II and 1.7% are grade III (4). As a result of advancement and increased application in magnetic resonance imaging (MRI) and computed tomography (CT), detection of asymptomatic meningiomas is becoming increasingly prevalent. Initial managements for asymptomatic meningiomas include observation, surgical resection and radiotherapy. However, the best treatment still remains controversial.

In most instances, many asymptomatic meningiomas has an indolent natural course. Only a small proportion of patients become symptomatic. In previous studies, 24%–92% of those meningiomas will increase in size under observation after a period of more than 4 years (1, 2, 5–10). Observation is a reasonable treatment option for those slow-growing and small asymptomatic tumors. But it may increase the treatment risk due to enlarged tumors and patient age. In a population-based analysis, the use of primary observation increased for smaller tumors (<2 cm), relative to surgery and radiation (11). In a recent meta-analysis, those tumors with larger size were likely to be symptomatic (12). Therefore, treatment should be recommended for large tumors, symptomatic tumors and those documented growth. Surgical resection is a better choice for tumor removal. But it is an invasive treatment, depends on tumor location and can cause significant morbidity (13). Gamma knife radiosurgery (GKRS) is more appealing than surgical resection, which is a less invasive treatment with a low morbidity.

There had been several publications reported the efficacy and safety of GKRS as primary or adjuvant treatment for asymptomatic meningiomas. These results showed a low tumor progression rate of 0-5.9%, and an acceptable rate of complications ranged from 2.4% to 20.5% after GKRS (7, 10, 14, 15). However, many patients in these studies had prior surgery or radiotherapy, peritumoral edema (PTE) or small tumors (<20 mm). The long-term outcomes of initial GKRS for asymptomatic meningiomas still need to be demonstrated. Therefore, we performed a single-center retrospective study consisting of patients with previous untreated, non-PTE and mainly large (≥20 mm) asymptomatic meningiomas.

## Methods

### Patients Population

**Figure 1:**
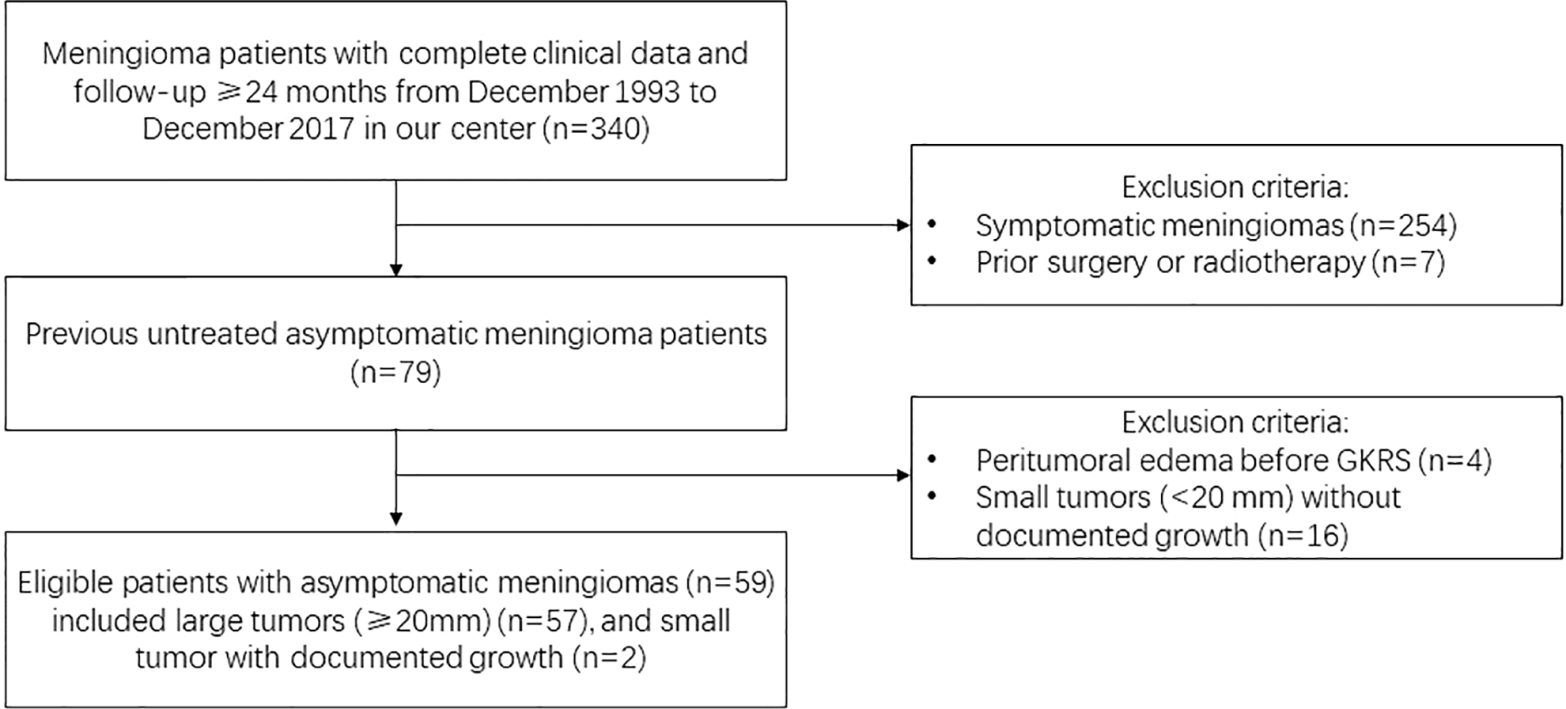
Study design with flow chart and initial GKRS criteria for large (≥20 mm) or documented growth asymptomatic meningiomas.

The medical records of meningioma patients from the single-center that underwent GKRS between December 1993 and December 2017 were retrospectively reviewed. There were 340 patients had complete clinical data and sufficient follow-up (≥24 months) in our hospital. Finally, 59 patients were included in this study. The GKRS inclusion criteria were shown in **Figure 1**. This study was approved by the institutional committee of the Second Affiliated Hospital of Guangzhou Medical University.

Inclusion criteria of this study: 1) MRI and/or CT findings suggested the diagnosis of meningioma, as interpreted by experienced clinicians and radiologists; 2) tumors with homogeneously contrast enhancement, dural tail and no perilesional edema; 3) the initial treatment was GKRS, no prior surgery or radiotherapy for the meningiomas; 4) no neurological symptoms or signs caused by the tumor; 5) large tumors (≥20 mm), or those small tumors with documented growth were included; 6) all of patients had complete medical records, at least one radiological and clinical evaluations with minimum follow-up of 24 months. Patients with neurofibromatosis type 2-associated meningiomas and PTE were excluded.

### Radiological and Clinical Evaluations

All of asymptomatic meningioma patients were required to take routine clinical and radiological follow-up evaluations at 6 months initially and thereafter yearly. The follow-up evaluations were evaluated by experienced clinicians and radiologists. Tumor volume was based on ABC/2 formula: (A) maximum tumor diameter on axial plane; (B) diameter perpendicular to (A), and (C) maximum height on sagittal/coronal plane (16). Tumor shrinkage was defined as a reduction in tumor size at least 20% in any diameter. Stable tumor was defined as a change in tumor size within 20% in any diameter. Tumor progression was defined as an increase in tumor size at least 20% in any diameter. New neurological symptoms or sighs after GKRS were recorded. Radiation related adverse effects and requiring treatment were reported.

### Gamma Knife Radiosurgery Technique

Before April 2014, all of the patients were treated with Leksell Gamma Knife Unit B. Leksell Gamma Knife Perfexion Unit (Elekta Instrument, Inc.) was used from April 2014 to the present. Patients were placed with Leksell stereotactic frame G and underwent stereotactic MR imaging with contrast through the entire brain for target delineation. GKRS plan was designed by a radiation oncologist, medical physicist, and neurosurgeon. 4 or 8 mm collimator was used for better conformality.

### Statistical Analysis

IBM’s SPSS (version 21.0) was used for statistical analyses. Univariate and multivariate analysis of risk factors associated with PTE after GKRS and symptom progression were carried out with Log-rank test statistics and a step forward likelihood ratio method of Cox proportional hazard models respectively. Kaplan-Meier curves were plotted for progression-free survival (PFS), PTE and symptom progression. Probability values <0.05 were defined as statistically significant.

## Results

### Patient Characteristics

Characteristics of patients were showed in **Table 1**. Fifty-nine patients who met inclusion criteria were included in this study. Of the 59 patients, 15 (25.4%) were male and 44 (74.6%) were female. The median age was 56 (range, 27–83) years. The median time of follow-up was 66.8 (range, 24.6–245.6) months. The median size was 22.0 (range, 15.7–57.0) mm. The median tumor volume was 3.9 (range, 2.5–6.8) ml. Two patients with tumor size <20 mm were documented with tumor growth on radiologic surveillance and enrolled in this study. The number of tumor size ≥30 mm was 12 (20.3%). The falx/parasagittal region was the most frequent tumor location, followed by the middle fossa (sphenoid/parasellar).

**Table 1:** Characteristics of 59 patients with 59 asymptomatic meningiomas and GKRS parameters.

Characteristic	Value
Male/Female, n (%)	15/44 (25.4/74.6)
Median age, (range), years	56 (27–83)
Median FU length, (range), months	66.8 (24.6–245.6)
Median tumor size, (range), mm	22.0 (15.7–57.0)
<20 mm, n (%)	2 (3.4)
< 30 mm, n (%)	45 (76.3)
≥30 mm, n (%)	12 (20.3)
Median tumor volume, (range), ml	3.9 (2.5–6.8)
Tumor location	
Convexity, n (%)	6 (10.2)
CPA, n (%)	8 (13.6)
Falx/parasagittal, n (%)	24 (40.7)
Cavernous sinus, n (%)	1 (1.7)
Tentorial, n (%)	3 (5.1)
Middle fossa (sphenoid/parasellar), n (%)	10 (16.9)
Intraventricular, n (%)	2 (3.4)
Frontobasal, n (%)	5 (8.5)
GKRS parameters	
Median margin dose, (range), Gy	13.0 (11.6–20.0)
Median maximum dose, (range), Gy	32.0 (25.1–50.0)
Median prescription isodose, (range), %	40 (35–55)

FU, follow up; GKRS, gamma knife radiosurgery; CPA, cerebellopontine angle.

All of patients underwent single fraction GKRS as initial treatment for meningiomas. The median margin dose was 13.0 (range, 11.6–20.0) Gy at a median prescription isodose 40% (range, 35%–55%). The median maximum dose was 32.0 (range, 25.1–50.0) Gy (**Table 1**).

### Radiological and Symptom PFS

**Figure 2:**
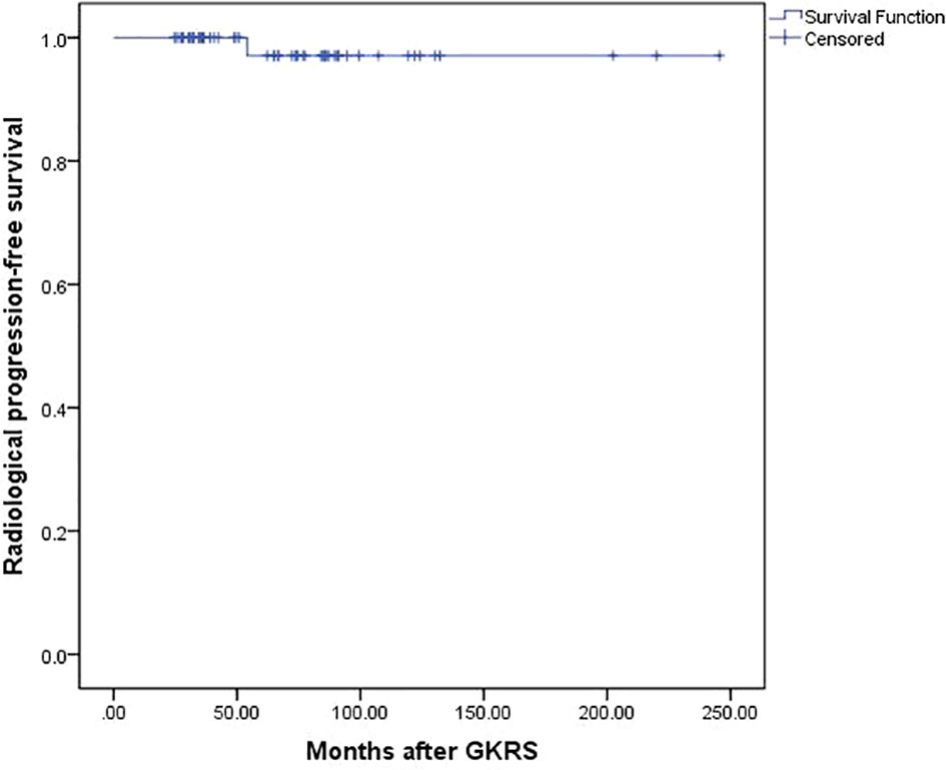
Kaplan-Meier curve of progression-free Survival for the entire series.

Overall, 58 patients (98.3%) were confirmed as tumor control after a median follow-up time of 66.8 (range, 24.6–245.6) months. Tumors shrunk in 35 patients (59.3%) and remained stable in 23 patients (39.0%). One patient (1.7%) experienced radiological progression at 54 months after GKRS. The PFS was 100%, 97%, and 97% at 3, 5, and 10 years, respectively (**Figure 2**) (**Table 2**).

**Table 2:** Outcomes of 59 patients with 59 asymptomatic meningiomas after initial GKRS.

Outcomes	value
Tumor control	
Shrinkage, n (%)	35 (59.3)
Stable, n (%)	23 (39.0)
Progression, n (%)	1 (1.7)
Complications after GKRS	17 (28.8)
Radiation-induced peritumoral edema	15 (25.4)
Symptomatic, n (%)	7 (11.9)
Asymptomatic, n (%)	8 (13.6)
Symptom unrelated with PTE	2 (3.4)
Memory loss	1
Cranial nerve deficit	1

**Figure 3:**
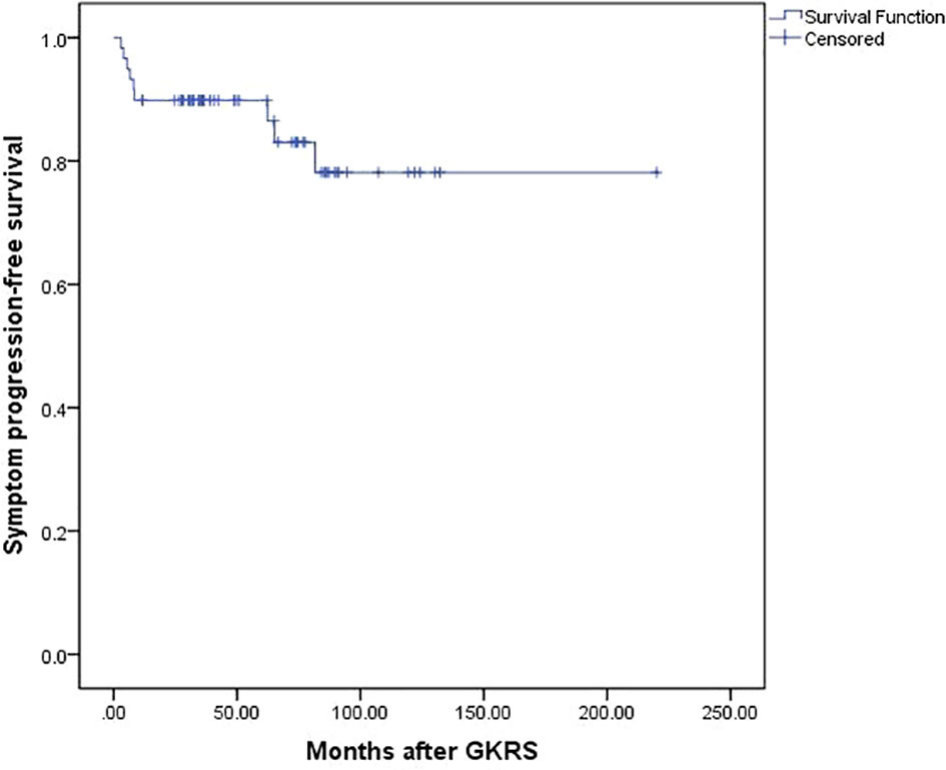
Kaplan-Meier curve of Symptom progression-free survival for the entire series.

The symptom PFS was defined as the time interval between the time of occurrence of new neurological symptoms or signs and the time of GKRS in this study. Nine patients (15.3%) occurred new neurological symptoms or signs after GKRS at a median time of 8.1 (range, 3.0–81.6) months. Of the nine patients, seven patients presented with new neurological symptoms or signs might be due to PTE. Another two patients presented with memory loss and cranial nerve deficit did not occur PTE after GKRS. The symptom PFS was 90% and 78% at 5 and 10 years, respectively (**Figure 3**) (**Table 2**).

### Complications After GKRS

**Figure 4:**
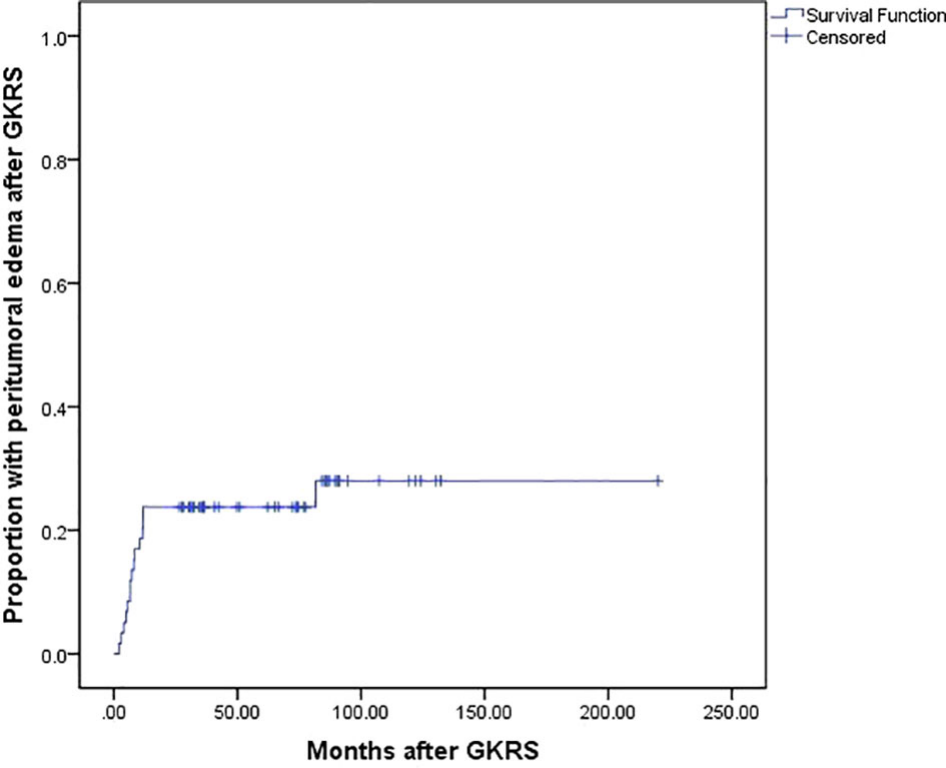
Kaplan-Meier curve of proportion with peritumoral edema after GKRS for the entire series.

Seventeen patients (28.8%) developed complications after GKRS, including 15 patients (25.4%) with PTE and 2 patients with symptom unrelated with PTE. The median time of PTE was 7.2 (range, 2.0–81.6) months. Of these PTE patients, eight patients (13.6%) were asymptomatic PTE. Another seven patients (11.8%) presented with symptomatic PTE. Among of them, one patient underwent surgical resection for severe PTE after GKRS (**Table 2**). The rate of PTE after GKRS was 14%, 24%, and 24% at 6, 12, and 60 months, respectively (**Figure 4**).

**Figure 5:**
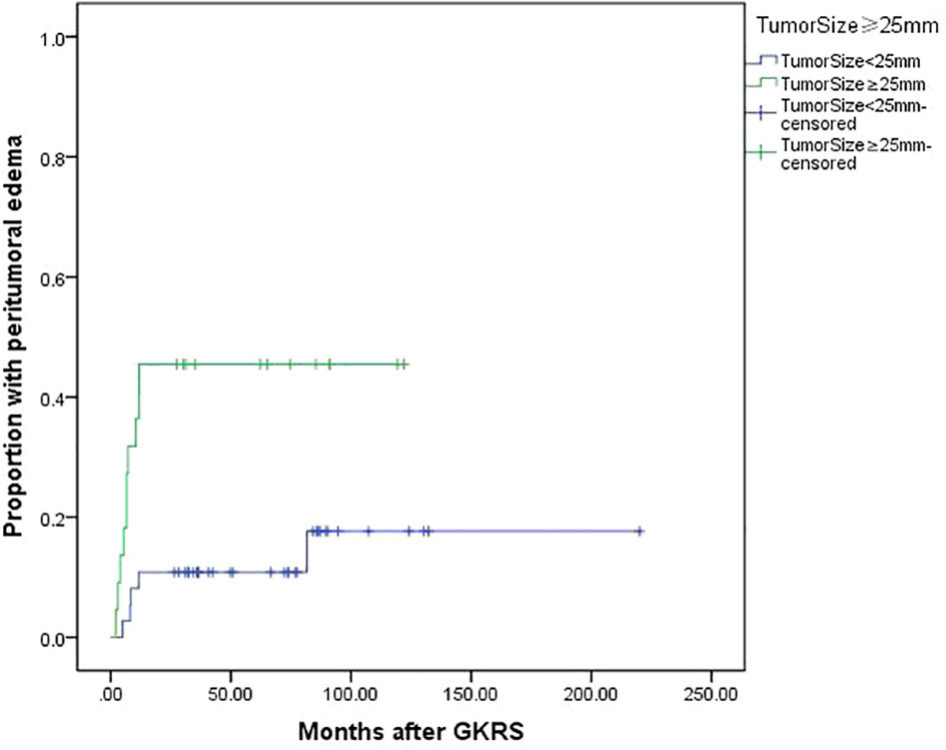
Kaplan-Meier curve of peritumoral edema after GKRS of tumor size ≥25 mm vs. <25 mm. Tumor size ≥25 mm showed a higher peritumoral edema rate after GKRS (p=0.004).

**Figure 6:**
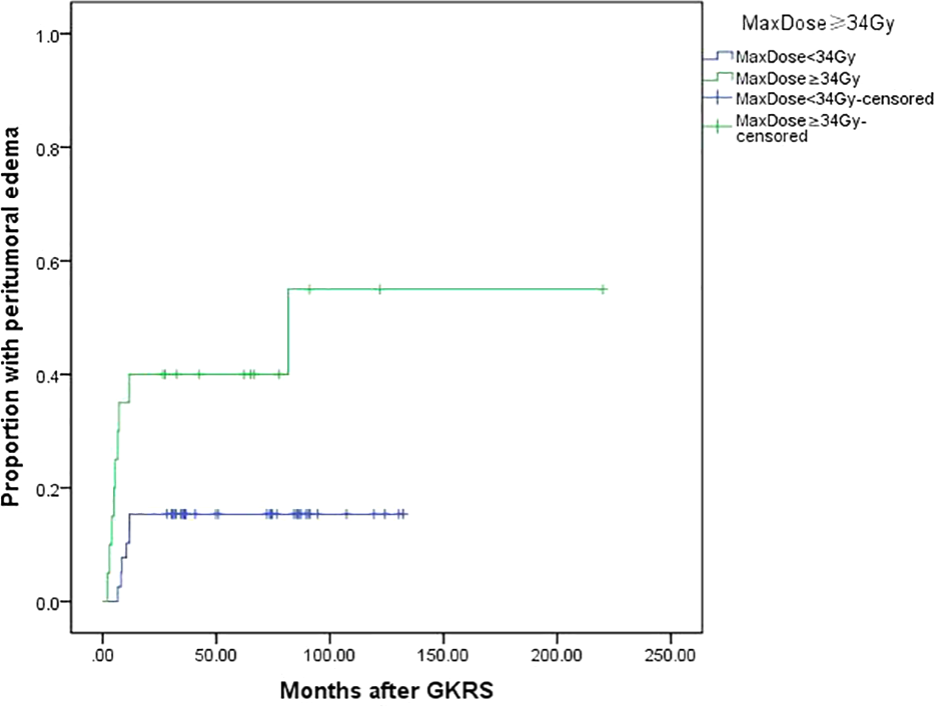
Kaplan-Meier curve of peritumoral edema after GKRS of maximum dose ≥34 Gy vs. <34 Gy. Maximum dose ≥34 Gy showed a higher peritumoral edema rate after GKRS (p=0.005).

Tumor size (≥25 mm) (p=0.004), tumor volume (≥5.3 ml) (p=0.038) and maximum dose (≥34 Gy) (p=0.005) were associated with PTE in univariate analysis. Tumor size (≥25 mm), maximum dose (≥34 Gy), tumor volume (≥5.3 ml) and tumor margin dose (≥14 Gy) were included in multivariate analysis. Only tumor size (≥25 mm) (**Figure 5**) and maximum dose (≥34 Gy) (**Figure 6**) were significantly associated with PTE [hazard ratio (HR)= 3.461, 95% confidence interval (CI)=1.157-10.356, p=0.026 and HR=3.067, 95% CI=1.068-8.809, P=0.037, respectively] (**Table 3**).

**Table 3:** Results of univariate and multivariate analysis for peritumoral edema after GKRS.

Variables	Peritumoral edema after GKRS			
	Univariate, p	Multivariate, p	HR	95% CI
Age (≥55 years)	0.935	NA	NA	NA
Sex (male vs female)	0.855	NA	NA	NA
Skull base vs non skull base	0.428	NA	NA	NA
Tumor margin dose (≥14 Gy)	0.100	0.477	NA	NA
TV ≥5.3 ml	0.038*	0.118	NA	NA
Tumor size (≥25 mm)	0.004*	0.026*	3.461	1.157–10.356
Maximum dose (≥34 Gy)	0.005*	0.037*	3.067	1.068–8.809

CI, confidential interval; HR, hazards ratio; TV, tumor volume; NA, not available.

*Statistically significant (P < 0.05).

## Discussion

### Natural History of Meningiomas

As many asymptomatic meningiomas has an indolent natural course. Understanding the natural history of them is of great importance to make appropriate management. Sughrue et al. (17) performed a systematic review of 675 patients with untreated meningiomas followed with serial MR imaging from 22 studies. The authors found initial tumor diameter of 2–2.5 cm with the rapid growth of >10% per year was prone to occur symptom progression at a rate of 42%. They also found initial tumor diameter of >2.5 and 3 cm went on to develop new or worsened symptoms 17% of the time. In a recent meta-analyses, Nakasu et al. (12) also found large initial maximum diameter were significantly associated with symptomatic progression. As a high risk of symptom progression in large tumors, these patients may gain benefit from earlier treatment before the occurrence of symptoms.

### Advantage and Limitation of Surgery

Surgical resection has advantages of rapid tumor removal and histopathologic analysis. Recent European Association of Neuro-Oncology guidelines indicates that surgery is the first option when treatment is indicated in meningioma of any WHO grade (18). However, surgical resection is an invasive treatment, depends on tumor location and can cause significant morbidity (12). In the study of Naslund et al. (19), 30-day complication rate was significantly higher among the asymptomatic cases. Besides, asymptomatic meningioma patients were less likely to work full time after surgical resection as compared with preoperative status. Yano et al. (20) reported older patients (>70 years) had a higher complication rate after surgical for asymptomatic meningiomas than younger patients (9.3% vs. 4.4%). For asymptomatic meningioma patients, surgical resection could make patients worse, at best unchanged (21). In the study of Jakola et al. (21), almost one in five patients developed subjective deterioration after surgery at a long-term follow up. Considering the potentially significant morbidity of surgery for asymptomatic meningiomas, surgical resection should be a more restrictive approach.

### Gamma Knife Radiosurgery for Asymptomatic Meningiomas

A less invasive procedure, a low mortality and better function protection are the advantages of GKRS. These make GKRS more attractive and a preferred choice for asymptomatic meningiomas. A systematic review, meta-analysis and practice guideline (22) from international stereotactic radiosurgery society (ISRS) recommended radiosurgery may be proposed as a primary treatment modality for an asymptomatic or mildly symptomatic meningioma. Some authors (7, 10, 14, 15) had reported GKRS for asymptomatic meningiomas had a low tumor progression rate of 0%–5.9% and symptom progression rate of 2.6%–18%, as well as an acceptable rate of complications. **Table 4** summarizes recent literature reporting GKRS for asymptomatic meningiomas. Jo et al. (7) reported 69 asymptomatic meningiomas underwent GKRS with mean follow up of 63.0 months. The mean tumor diameter was 17.3 (range, 7–31 mm). The tumor control rate was 100% with median margin dose of 14.5 (range, 12–20) Gy. Transient complications developed in 39.1% of patients. There was no patient developed permanent neurologic deficit. Salvetti et al. (14) reported a tumor progression rate of 2.4% and permanent neurological deficit rate of 2.4% in 42 asymptomatic meningiomas treated with GKRS with a median margin dose of 15 (10–18) Gy. In this study, 23.8% of patients were small tumor (<20 mm). The proportion of prior surgical resection and radiotherapy were 45.2% and 4.8% respectively. Kim et al. (10) reported efficacy of proactive GKRS (n=153) in asymptomatic meningiomas compared with natural course (n=201). The radiological PFS rates in GKRS and observation group were 94.4% and 38.5% at 5 years, and 88.5% and 7.9% at 10 years, respectively. Adverse events developed in 13.3% of patients treated with GKRS, including 0.5% of patients requiring surgery due to severe edema after GKRS. 15.7% of patients in GKRS group presented with peritumoral edema before GKRS, which was associated with adverse events after GKRS (HR 4.885, 95% CI 1.660–14.376, P=0.004). In the study of Gupta et al. (15), 117 patients with 122 asymptomatic tumors were treated with GKRS with median dose of 14 (10–25) Gy. Thirty-nine percent of patients had prior surgical resection. Patients who presented with peritumoral edema were excluded. The radiological and clinical PFS were 97% and 86% at 5 years, 94.4% and 70% at 10 years, respectively. Neurological complications developed in 18% of patients.

**Table 4:** Literature review of GKRS for asymptomatic meningiomas.

Study	No. of patients	Median margin dose, (range), Gy	Median isodose line (range), %	Imaging follow-up, (months)	Tumor progression	Symptom progression	complications
Salvetti et al. (14)	42	15 (10–18)	48 (30–55)	Median 59	2.4%	4.8%	2.4% (permanent neurological deficits)
Jo et al. (7)	69	14.5 (12–20)	NA	Mean 63.0	0	NA	39.1% (transient complications)
Kim et al. (10)	153	14 (10–22)	50 (15–60)	Median 53	5.9%	2.6%	13.3% (mainly transient complications)
Gupta et al., (15)	117	14 (10–25)	50 (10–55)	Median 53	2.6%	18%	20.5%
Current study	59	13.0 (11.6–20.0)	40 (35–55)	Median 66.8	1.7%	15.3%	28.8%

GKRS, gamma knife radiosurgery.

In the current study, 59 asymptomatic meningiomas were treated with initial GKRS. Patients who presented with peritumoral edema were excluded. Two patients with small tumors (<20 mm) documented with tumor growth under radiological surveillance were included. One patient (1.7%) experienced radiological progression. The PFS was 100%, 97% and 97% at 3, 5, and 10 years, respectively. Nine patients (15.3%) occurred new neurological symptoms or signs after GKRS. The symptom PFS was 90% and 78% at 5 and 10 years respectively. Fifteen patients (25.4%) occurred PTE after GKRS.

### Peritumoral Edema and Related Factors

PTE is the most common adverse radiation effect after GKRS for meningiomas, which accounts for 15%–28% (23–30). Several clinical risk factors associated with PTE after GKRS had been reported, including sagittal sinus occlusion, parasagittal location, large tumor volume, presence of pretreatment edema, high radiation dose, high grade histology, and hemispheric tumor location (22–29). Kollová et al. (25) found significant risk factors associated with PTE after GKRS included tumor margin dose greater than 16 Gy and tumor volume greater than 10 cm^3^. Hoe et al. (31) reported 15.3% of patients developed new or increased PTE after GKRS, 8.8% of patients were symptomatic PTE. Pre-treatment PTE, hemispheric tumor location and tumor volume larger than 4.2 cc were associated with PTE.

PTE at diagnosis had been reported to be associated with high-grade meningiomas (32). Therefore, patients with pretreatment PTE were excluded in this study. The result in this study was similar with previous studies. 25.4% of patients developed PTE after GKRS with median time of 7.2 (range, 2.0–81.6) months. Of this patients, seven cases (11.9%) were symptomatic PTE. One patient received surgical resection for severe PTE. Univariate and multivariate analysis indicated large tumor size (≥25 mm) and maximum radiation dose (≥34 Gy) were associated with PTE after GKRS.

### Study Limitations

In this study, we restricted certain patients (large tumors, no PTE, and small tumors documented with growth) to undergo initial GKRS. This was a single-center retrospective study with selection and treatment biases. All of patients were diagnosed based on MRI or CT, and did not receive surgical resection before GKRS. Therefore, the histological diagnosis was not available. Besides, the number of patients in our study was small, which might affect statistical analysis. Finally, there was no control group to compare with those with natural course or treated with surgery.

## Conclusion

In this single-center retrospective study, the results supported the efficacy and safety of initial GKRS for asymptomatic meningiomas. 98.3% of patients were under tumor control after GKRS. 15.3% of patients occurred new neurological symptoms or signs. For those large asymptomatic tumors or small tumors documented growth, initial GKRS could prevent tumor progression with an acceptable rate of complications. GKRS could be an alternative treatment modality for selected asymptomatic meningiomas.

## Data Availability Statement

The raw data supporting the conclusions of this article will be made available by the authors, without undue reservation.

## Ethics Statement

The studies involving human participants were reviewed and approved by The Second Affiliated Hospital of Guangzhou Medical University Institutional Review Board. The patients/participants provided their written informed consent to participate in this study.

## Author Contributions

LW, CP, XY, HY, LC, and YD collected the data. JF, LW, CP, and XY analyzed the data. JY, JF, and LW wrote the paper. JY conceived and designed the study. All authors contributed to the article and approved the submitted version.

## Funding 

This work was supported by National Key Research and Development Project (grants number: 2017YFC0113700); National Natural Science Foundation of China (grants number: 81902928). China Postdoctoral Science Foundation Funded Project (2018M643343); Doctoral initiation project of Second Affiliated Hospital of Guangzhou medical university (010G271104).

## Conflict of Interest

The authors declare that the research was conducted in the absence of any commercial or financial relationships that could be construed as a potential conflict of interest.
